# Therapeutic response and prognostic factors of 14 dogs undergoing transcatheter arterial embolization for hepatocellular masses: A retrospective study

**DOI:** 10.1111/jvim.16746

**Published:** 2023-05-24

**Authors:** Yuta Kawamura, Hiroki Itou, Akitomo Kida, Hiroki Sunakawa, Moe Suzuki, Kenji Kawamura

**Affiliations:** ^1^ Kawamura Animal Hospital Niigata City Japan; ^2^ Department of Radiology, Division of Diagnostic Radiology, Faculty of Medicine Yamagata University Iida‐Nishi Japan

**Keywords:** canine, hemoabdomen, interventional oncology, median survival time, tumor reduction rate

## Abstract

**Background:**

Information regarding the therapeutic effect and outcome of transcatheter arterial embolization (TAE) for hepatic masses is limited in veterinary medicine.

**Hypothesis/Objectives:**

To analyze the therapeutic response, outcome (overall survival), and their predictors in dogs that underwent TAE for primary hepatocellular masses. We hypothesized that larger pre‐TAE tumors would be associated with worse outcomes.

**Animals:**

Fourteen client‐owned dogs.

**Methods:**

Retrospective study. Medical records between 1 September 2016 and 30 April 2022 were reviewed to identify dogs treated with TAE for hepatic masses diagnosed as hepatocellular origin by cytological or histopathological examination. Computed tomography images were compared before and after TAE. The univariate Cox proportional hazards test was performed to assess the associations between variables and survival. Univariate linear regression analysis was performed to assess the associations between variables and the tumor reduction percentage: ([post‐TAE volume − pre‐TAE volume]/pre‐TAE volume) × 100.

**Results:**

The median survival time was 419 days (95% confidence interval, 82‐474). History of intra‐abdominal hemorrhage (*P* = .03) and pre‐TAE tumor volume/body weight (*P* = .009) were significantly associated with overall survival. The mean reduction percentage was −51% ± 40%. Pre‐TAE tumor volume/body weight ratio (cm^3^/kg; *P* = .02, correlation coefficient = 0.704) was significantly correlated with the volume reduction percentage.

**Conclusions:**

History of intra‐abdominal hemorrhage and large pre‐TAE tumor volume/body weight ratio could be predictive factors for adverse outcomes after TAE. Pre‐TAE tumor volume/body weight ratio could be a predictive factor for therapeutic effect.

**Abbreviations**
CI: confidence interval
CT: computed tomography
DSA: digital subtraction angiography
FNB: fine needle biopsy
HR: hazard ratio
MST: median survival time
TACE: transcatheter arterial chemoembolization
TAE: transcatheter arterial embolization

## 1 INTRODUCTION

Primary liver tumors are relatively rare in dogs, accounting for 0.6% to 1.3% of all tumors in dogs.^1^ The most common group of primary hepatic masses in dogs are hepatocellular masses such as hepatocellular carcinoma, hepatocellular adenoma, and nodular hyperplasia.^2^ Hepatocellular masses account for 52% of primary liver masses.^3^ Regardless of the histological malignancy, they can cause various clinical signs such as gastrointestinal disease, jaundice, ascites, and intra‐abdominal hemorrhage by increasing in size within the abdominal cavity.^3^ Surgery is the first‐line treatment for these conditions, but symptomatic or palliative treatment is the main treatment for dogs that cannot undergo surgery in veterinary medicine.^4^, ^5^

Transcatheter arterial embolization (TAE) is a procedure in which embolizing material is injected through a catheter into the blood vessel that feeds the tumor, causing the tumor to undergo ischemic necrosis.^6^, ^7^, ^8^ Whereas normal liver parenchyma obtains approximately 80% of the blood supply through the portal vein, primary hepatocellular masses obtain most of the blood supply from the hepatic artery.^9^, ^10^, ^11^, ^12^, ^13^ Therefore, arterial embolization via the hepatic artery is minimally invasive and has a high antitumor effect.

In human medicine, TAE could be used for curative or palliative purposes, such as preventing bleeding or shrinking tumors. TAE is an important component of the treatment of unresectable hepatocellular masses and is one of the standard therapies.^6^, ^8^, ^14^, ^15^ However, in veterinary medicine, studies on TAE consist mainly of case reports with few comprehensive studies, leading to an inability to assess the effectiveness of this intervention in veterinary patients.^16^, ^17^, ^18^, ^19^

The purpose of this study was: (a) to analyze the outcome (overall survival) and predictive factors for outcomes in dogs with hepatocellular masses undergoing TAE, and (b) to analyze the tumor reduction percentage calculated using computed tomography (CT) and predictive factors for therapeutic effect. The authors hypothesized that larger pre‐TAE tumors would be associated with a worse prognosis than smaller tumors.

## 2 MATERIALS AND METHODS

### 2.1 Case selection

Fourteen dogs with hepatic masses of hepatocellular origin as confirmed by cytology or histopathology that underwent TAE between 1 September 2016 and 30 April 2022 in our hospital were included in this retrospective study. The dogs were staged with standard techniques including physical examination, thoracic CT examination, abdominal CT angiography, CBC, serum biochemistry profile, and urinalysis. All dogs were judged to be high‐risk surgical cases by surgeons because their masses involved large vessels, such as the caudal vena cava, or were located at the base of the liver lobe, or their owners declined open surgery because they were aged or had comorbidities. All owners were informed about the potential risks and complications of TAE.

### 2.2 Medical records review

Medical records were reviewed, and data recorded included signalment (age, breed, and sex), body weight, presenting clinical signs at diagnosis of hepatic masses, history of jaundice, abdominal effusion and abdominal hemorrhage, diagnostic imaging findings, tumor volume measurements, biopsy and cytologic findings, TAE procedural information and schedule, procedural complications, postprocedural complications, and survival times. Intra‐abdominal bleeding was defined as grossly red fluid in the peritoneal cavity with a PCV of 5% or more. These data were analyzed as prognostic factors of outcomes for the primary endpoint and predictive factors for the secondary endpoint.

### 2.3 Triple‐phase computed tomography angiography

A triple‐phase CT examination of the abdomen was performed in all dogs. For CT examination, a 16‐row multislice CT scanner (Brivo CT385; GE Healthcare, Fairfield, NJ) was used. The dogs underwent general anesthesia for CT examination. Dogs were premedicated with butorphanol tartrate (0.25 mg/kg, IV) and atropine sulfate (0.01 mg/kg, IV), and induction of anesthesia was achieved with IV administration of propofol (3 mg/kg) after pre‐oxygenation. After induction, endotracheal intubation was performed, and anesthesia was maintained with inhaled isoflurane (1.6%‐2.0%). Ephedrine hydrochloride (1 mg/kg, IV bolus as necessary) was used to maintain blood pressure. Iohexol contrast medium (300 mgI/mL; Omnipaque 300; Daiichi Sankyo, Tokyo, Japan) was injected (2.0 mL/kg, iv) at 0.1 mL/kg/s. The images were taken at the times of the arterial phase (20 seconds), portal phase (40 seconds), and equilibrium phase (120 seconds); the settings used were 120 kVp, 200 mA, and 1.2‐mm collimation. Imaging software (OsiriX; Pixmeo, Bernex, Switzerland) was used to reconstruct the three‐dimensional CT angiography, identify the feeding vessels to the tumor, and determine whether TAE could be performed.

In addition, for cases in which biopsy had not been performed, fine needle aspiration biopsy, Tru‐Cut needle biopsy, or laparoscopic biopsy was performed at the same time as CT examination.

### 2.4 TAE procedure

TAE was performed within 1 month from the CT examination, and all procedures were performed by the same surgeon.

For the TAE procedure, cefovecin sodium (8 mg/kg, sc) was administered as a preoperative antibiotic. Atropine sulfate (0.01 mg/kg, iv), midazolam (0.25 mg/kg, iv), and butorphanol (0.25 mg/kg, iv) were administered as preanesthetic medications. Anesthesia was induced with propofol, which was slowly administered intravenously to effect. After induction, endotracheal intubation was performed, and anesthesia was maintained with inhaled isoflurane (1.4%‐2.2%). Ephedrine hydrochloride (1 mg/kg, IV bolus as necessary) was used to maintain blood pressure. The dog was placed in the supine position, and the right or left groin was shaved and disinfected.

**FIGURE 1:**
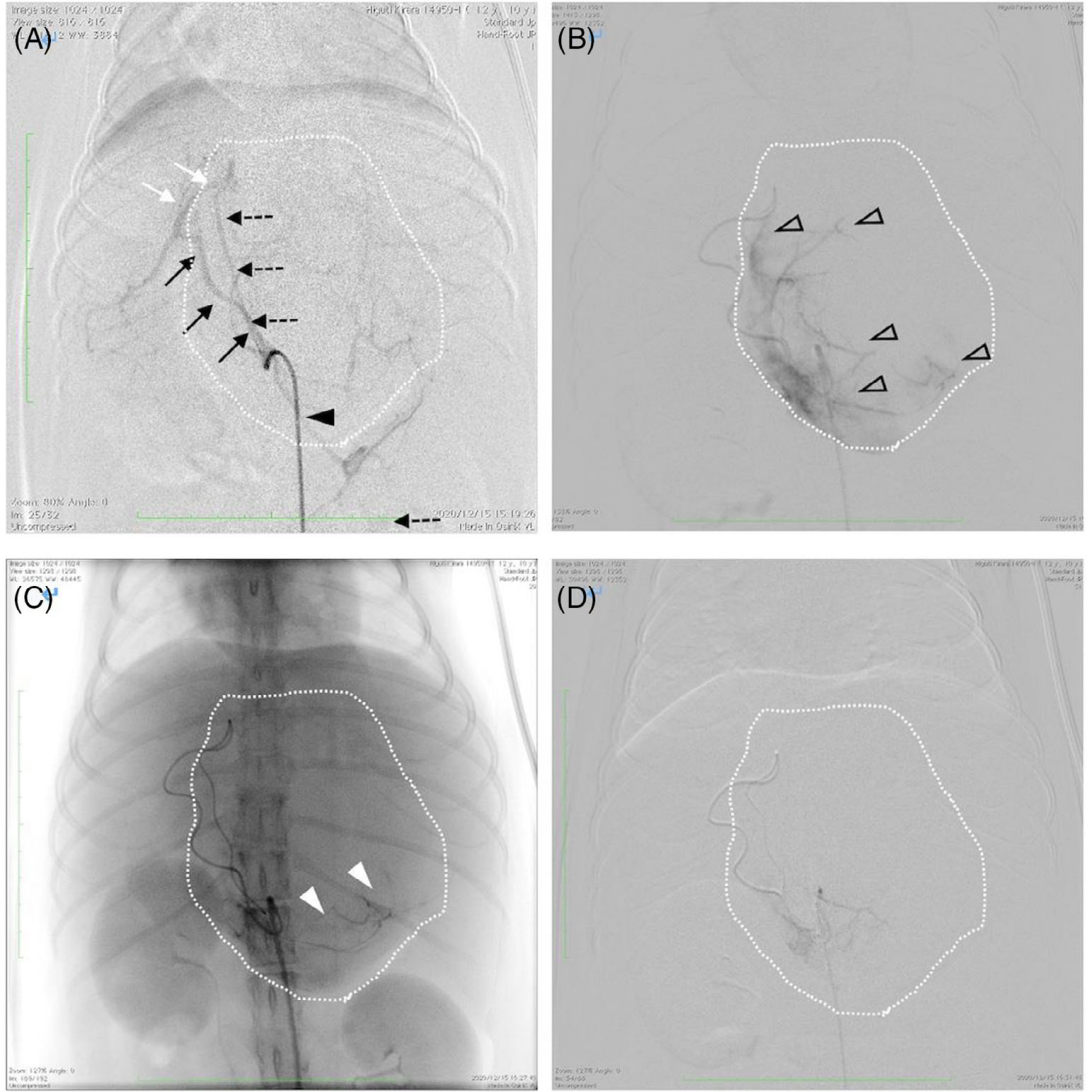
Fluoroscopic images obtained during the TAE procedure. The dog's head is at the top of each ventrodorsal image. The dotted white line represents the outline of the mass. (A) Angiography from the celiac artery. The black arrowhead indicates a guiding catheter, the tip of which is located in the celiac artery. The black arrows indicate the common hepatic artery, the white arrows indicate the left hepatic artery, and the black dotted arrows indicate the tumor‐feeding vessel. (B) Angiography from the tumor‐feeding vessel. Perfusion of the entire tumor was confirmed (hollow arrowheads). (C) Injection of an embolic agent through the feeding vessel. The white arrowheads indicate the contrast agent mixed with the embolic agent. (D) Angiography after TAE shows decreased tumor perfusion and no nontarget embolization. TAE, transcatheter arterial embolization.

The following procedure was performed using a flat panel‐type X‐ray fluoroscope (Cios Fusion; Siemens, Munich, Germany). A 4‐ or 5‐Fr short sheath introducer (Radifocus Introducer IIH; Terumo, Tokyo, Japan) was inserted into the right (or left) femoral artery using a cut‐down technique. A 4.2‐Fr RC (4.2‐Fr RC‐S shape for small dogs) guiding catheter (Excellent EN catheter; Hanaco Medical, Saitama, Japan) was then inserted; RC guiding catheters have a hook‐shaped tip and are suitable for isolating the celiac artery. The catheter was advanced beyond the celiac artery in the abdominal aorta, and the celiac artery was isolated by slowly pulling back with the catheter tip directed caudally. The catheter tip was then placed proximal to the celiac artery and angiography was performed to confirm the anatomy of the hepatic artery and feeding vessels of the tumor (Figure 1A). A 1.7‐Fr microcatheter (Estream 1.7; TORAY, Tokyo, Japan) was then inserted through the 4.2‐Fr guiding catheter. Referring to the blood vessel identified on angiography, the common hepatic artery was isolated, the microcatheter was advanced to the front of the branch, and angiography was performed again to identify tumor‐feeding vessels. An angled 0.014‐in. microguidewire (Cross Winder; TORAY, Tokyo, Japan) was inserted, advanced into the feeding vessel, and traced with a microcatheter. Angiography around the vicinity of the tumor revealed intense staining of the entire tumor (Figure 1B). At this time, the microcatheter was inserted as far as possible to avoid embolization of the normal hepatic parenchyma. As an embolization agent, fine grains of gelatin sponge (Gelpart 1‐mm grain; Nippon Kayaku, Tokyo, Japan) were used in 10 dogs, and bead‐shaped embolization materials (Embosphere 100‐300 μm grain; Merit Medical Systems, South Jordan, UT) were used in 4 dogs.

In the dogs receiving Gelpart, the particle size was reduced using the following method before administration: After injecting 10 mL of iohexol contrast medium (300 mgI/mL) into a vial containing Gelpart, mixing was performed by inversion, and the mixture was aspirated into a 2.5‐mL syringe; this syringe and another empty 2.5‐mL syringe were connected to a 3‐way stopcock at a 90° angle. The particle size was reduced by reciprocating the inner cylinder of the syringe 20 times with the 3‐way stopcock open. It is known that pumping the syringe 20 times will result in a particle diameter of 0.5 mm.^20^

In the dogs receiving Embosphere, the particles were adjusted using the following method before administration: The total volume in the Embosphere syringe was matched with the same volume of iohexol contrast medium (300 mgI/mL), which resulted in a 50% microsphere/saline and 50% contrast solution. All air was removed from the syringe. To evenly suspend the Embosphere microsphere/contrast solution, the syringe was gently inverted several times.

The presence of the microcatheter tip in the target vessel was reconfirmed with a test injection of contrast agent. After confirmation, the embolization agent was slowly injected into the target vessel using a 1‐mL syringe under fluoroscopic guidance (Figure 1C). Care was taken to prevent the flow of the embolization agent into vessels other than the target vessel. Blood flow decreased as embolization progressed. When the blood flow began to stagnate, the embolization agent remaining in the microcatheter was pushed out with physiological saline. Selective angiography was then performed to confirm the complete disappearance of the tumor stain (Figure 1D). If tumor stains remained, additional embolization agent was injected until they disappeared. The microcatheter was then withdrawn, and angiography was performed from the common hepatic artery to check for collateral vessels feeding the tumor. If collateral vessels were found, they were selectively catheterized and embolized. The 4.2‐Fr RIM‐S guiding catheter and sheath were then removed. The puncture site of the femoral artery was closed with 6‐0 polydioxanone suture (PDS; Ethicon, Raritan, NJ), and the skin of the inguinal region was closed routinely.

### 2.5 Postoperative course

Daily monitoring of body temperature, CBC, biochemical tests (alkaline phosphatase, alanine aminotransferase, aspartate aminotransferase, total bilirubin, blood urea nitrogen, creatinine, phosphate, calcium, and electrolyte levels), and abdominal ultrasonography was performed postoperatively to check for complications such as post‐embolism syndrome and tumor lysis syndrome. During hospitalization, administration of lactated Ringer's solution (3‐5 mL/kg/h, iv) and symptomatic treatment were performed for mild diarrhea and inappetence, which are considered to be post‐embolism syndromes, until the condition improved.

### 2.6 Endpoints

#### 2.6.1 Primary endpoint

Overall survival, an indicator of prognosis, was set as the primary endpoint of this study. Survival was defined as the time from the date of TAE to death.

#### 2.6.2 Secondary endpoints

Tumor reduction, an indicator of the therapeutic response, was set as the secondary endpoint. In these dogs, only the reduction in tumor volume after the first TAE was included in the analysis. The tumor volume at each time point was measured from the CT images before TAE and 1 to 2 months after TAE, and the reduction percentage was obtained using the formula: ([post‐TAE volume − pre‐TAE volume]/pre‐TAE volume) × 100.

**FIGURE 2:**
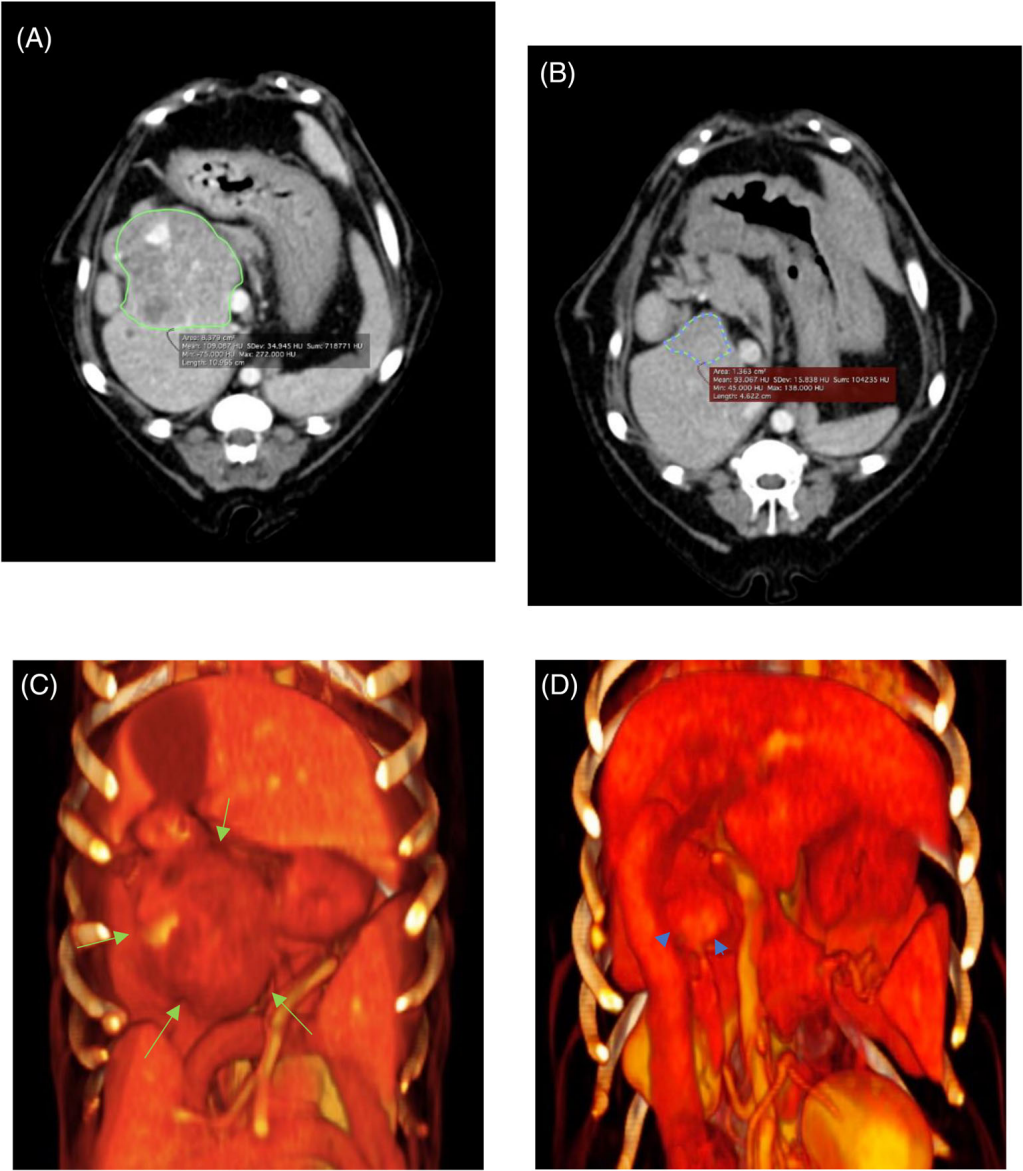
Reduction ratio calculation using computed tomography images. (A) Pre‐transcatheter arterial embolization (TAE) mass contour (green line). (B) Post‐TAE mass contour (green/blue dotted line). (C) Pre‐TAE mass three‐dimensional volume rendering (green arrow). (D) Post‐TAE mass three‐dimensional volume rendering (blue arrowhead).

OsiriX was used for image analysis and tumor reduction percentage calculation. Tumor volume measurement was performed by manually surrounding the contour of the tumor using a horizontal section of a CT image and using the volume measurement function (Figure 2). An automatic contour complement function was used when the number of slices was large.

### 2.7 Statistical analysis

EZR^21^ was used as statistical analysis software. *P* < .05 was considered to be statistically significant in each analysis.

The overall survival was analyzed by the log‐rank test. In addition, the univariate Cox proportional hazards test was used to analyze various pre‐defined prognostic factors. The hazard ratio (HR) was estimated using a 95% confidence interval (CI). The predictors included the following: dog factors such as age and body weight; tumor factors such as liver segment, pre‐TAE tumor volume, and pre‐TAE tumor volume divided by body weight (pre‐TAE tumor volume/body weight ratio); history of signs of gastrointestinal disease, ascites, weight loss, intra‐abdominal bleeding, or jaundice; and treatment factors such as whether the embolization agent was Gelpart or Embosphere.

Univariate linear regression analysis was performed to analyze the relationship between the tumor reduction percentage and various predictors. This univariate model was performed with the tumor reduction percentage as the dependent variable and the aforementioned predictors as independent variables. In addition, to examine the relationship between shrinkage and survival, a log‐rank test was performed on groups that had shrinkage above and below the median.

## 3 RESULTS

### 3.1 Case characteristics

Breeds represented in this study were toy poodle in 4 cases, miniature dachshund in three cases, shih tzu in two cases, and beagle, Shiba Inu, border collie, Yorkshire terrier, and Chihuahua in one case each. The average age was 12.1 ± 1.6 years, and the average weight was 7.3 ± 4.0 kg. There were two intact males, two castrated males, three intact females, and seven spayed females. Tumors were located in the right hepatic segment in six cases, middle hepatic segment in one case, and left hepatic segment in seven cases (Table 1). All dogs were discharged 2 to 5 days after TAE except 1 dog that died of tumor lysis syndrome the day after TAE. Ten dogs underwent follow‐up CT scans 1 to 2 months after TAE (53.7 days on average). Of these 10 dogs, two underwent a second TAE using the same embolic material ~1 to 1.5 years after the first TAE because the tumors began to enlarge again. The results of blood tests before and after TAE are presented in Table 2. In all dogs, the first TAE was performed within 1 month of staging. Six dogs were still alive at the end of the study period and eight dogs had died.

**TABLE 1:** Characteristics of 14 dogs undergoing TAE.

Category	Number of dogs
Breed	
Toy poodle	4
Miniature dachshund	3
Shih tzu	2
Beagle	1
Shiba	1
Border collie	1
Yorkshire terrier	1
Chihuahua	1
Sex	
Intact male	2
Castrated male	2
Intact female	3
Spayed female	7
Age (years)	
Median (range)	12.5 (9.3‐14.3)
Body weight (kg)	
Median (range)	5.8 (3.2‐15.9)
Hepatic segment	
Right	6
Middle	1
Left	7
Tumor volume (cm^3^)	
Median (range)	184.6 (22.8‐807.3)
Tumor volume / body weight ratio (cm^3^/kg)	
Median (range)	28.8 (4.9‐107.9)
History of signs of gastrointestinal disease	
Yes	9
No	5
History of abdominal effusion	
Yes	6
No	8
History of weight loss	
Yes	6
No	8
History of hemoabdomen	
Yes	5
No	9
History of jaundice	
Yes	1
No	13
Embolic agent	
Gelpart	10
Embosphere	4
Biopsy	
Hepatocellular mass (cytology)	8
Hepatocellular adenoma	2
Hepatocellular carcinoma	4

Abbreviation: TAE, transcatheter arterial embolization.

**TABLE 2:** Blood test results before and after TAE in the dogs of this study.

	n	Median	Range
ALT (IU/L)			
Pre‐TAE	14	236	70‐1891
1 day	14	1942	81‐4332
1 week	13	683	11‐2460
4 weeks	11	130	34‐1056
ALP (IU/L)			
Pre‐TAE	14	1157	210‐7704
1 day	14	2327	345‐8685
1 week	13	2001	360‐5691
4 weeks	11	864	192‐1968
TBIL (mg/dL)			
Pre‐TAE	13	0.2	0.1‐1.0
1 day	12	0.2	0.1‐1.2
1 week	10	0.3	0.1‐0.4
4 weeks	6	0.2	0.1‐0.5
White blood cell count (/μL)			
Pre‐TAE	14	10 400	7500‐36 900
1 day	13	16 000	9200‐45 000
1 week	7	11 100	5200‐21 400
4 weeks	8	10 200	5800‐15 200
Hematocrit (%)			
Pre‐TAE	14	35.8	24.7‐44.9
1 day	13	31.2	20.2‐46.9
1 week	7	25.9	21.9‐37.3
4 weeks	8	37.8	25‐47.3

Abbreviations: ALP, alkaline phosphatase; ALT, alanine aminotransferase; TAE, transcatheter arterial embolization; TBIL, total bilirubin.

### 3.2 Primary endpoint

**FIGURE 3:**
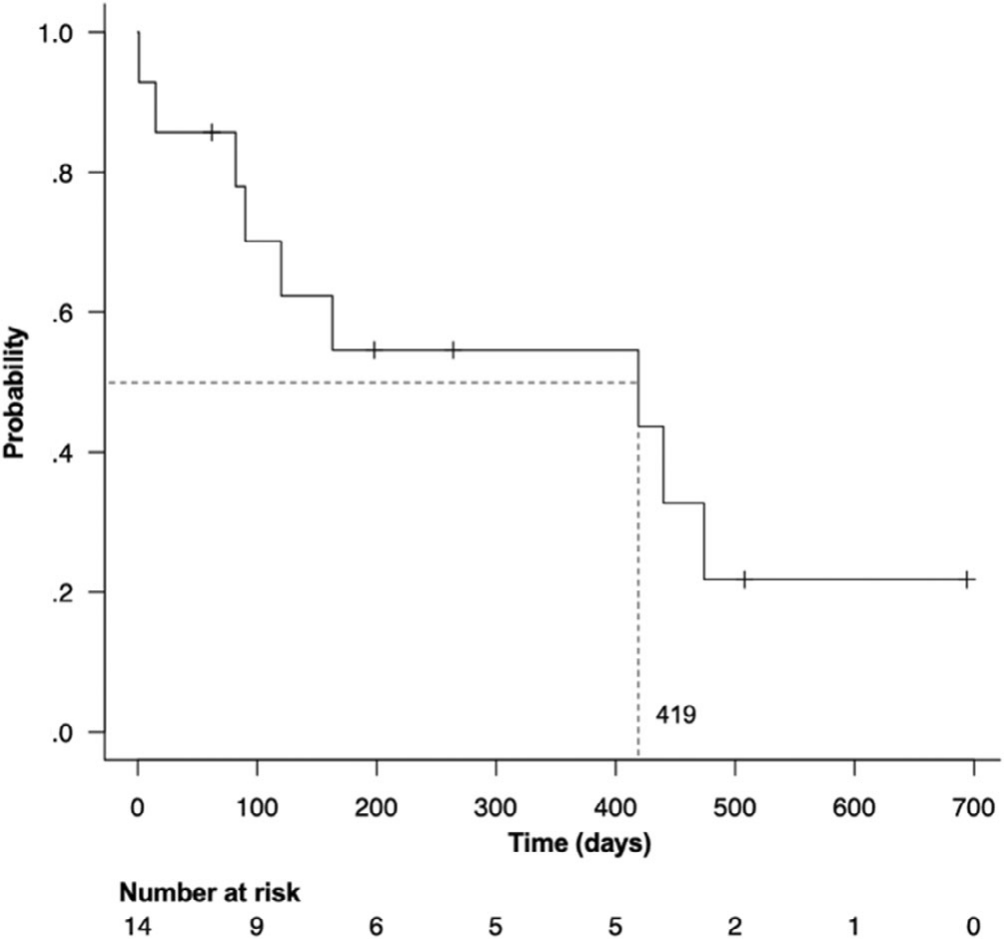
Kaplan‐Meier survival curve of 14 dogs undergoing transcatheter arterial embolization for hepatocellular masses. The median survival time is 419 days.

**FIGURE 4:**
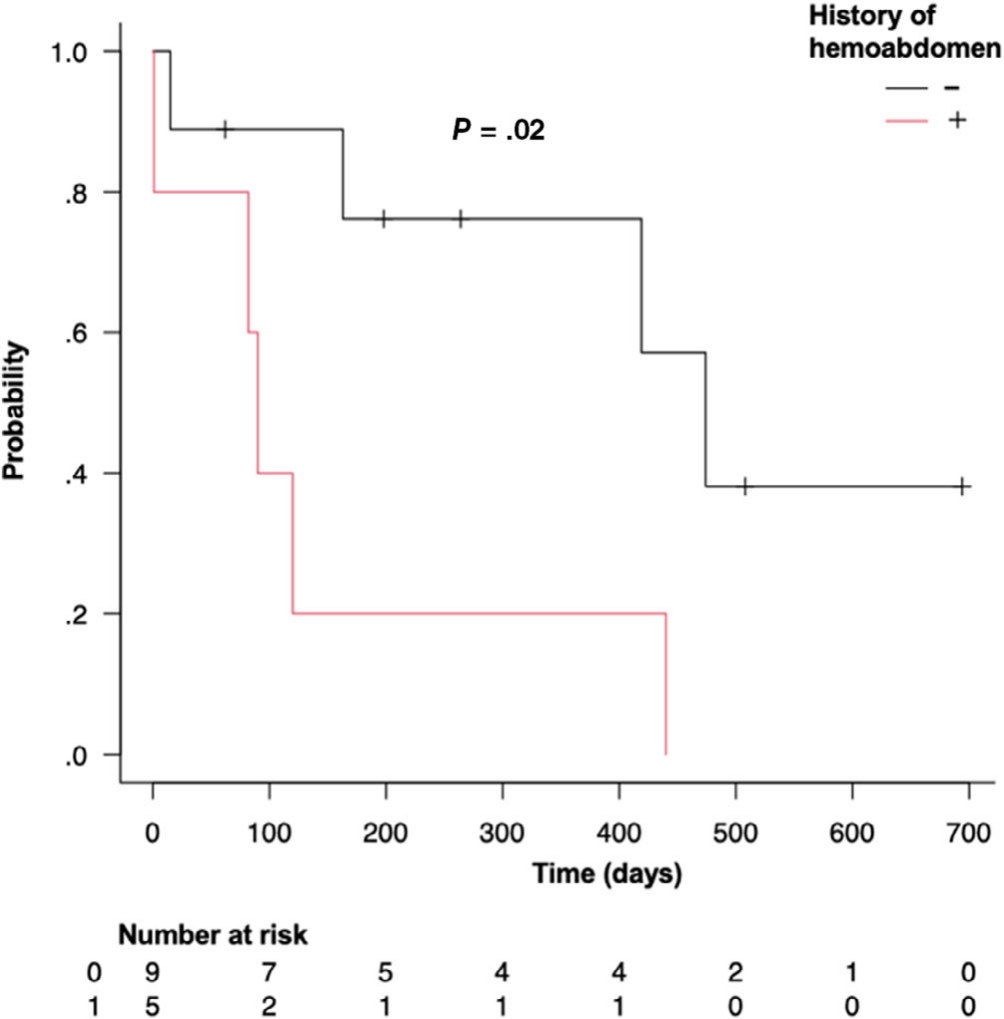
Kaplan‐Meier survival curves of 14 dogs undergoing transcatheter arterial embolization for hepatocellular masses with (red) and without (black) a clinical history of hemoabdomen.

In the survival analysis of all 14 dogs, the median survival time (MST) was 419 days (95% CI: 82‐474, range: 1‐694) and the 1‐year survival rate was 55% (Figure 3). Prognostic factor analysis using a univariate Cox proportional hazards test showed that a history of intra‐abdominal bleeding was a significant predictive factor for outcome (HR: 4.909, 95% CI: 1.135‐21.22, *P* = .03; Table 3). Dogs with a history of intra‐abdominal bleeding had a significantly shorter survival time than those without (Figure 4). Pre‐TAE tumor volume/body weight ratio was also a significant predictive factor for outcome (HR: 1.058, 95% CI: 1.014‐1.103, *P* = .009). The results showed that survival was significantly shorter for dogs with higher tumor volume/body weight ratios than for those with lower ratios (Table 3).

**TABLE 3:** Univariate Cox proportional hazards model for predicting outcome in 14 dogs undergoing TAE for hepatocellular masses.

Variable	Hazard ratio (95% confidence interval)	*P* value
Dog factors		
Age (per 1 year)	1.006 (0.974‐1.038)	.72
Weight (per 1 kg)	0.959 (0.796‐1.156)	.66
Tumor factors		
Hepatic segment (vs left)		
Center	NA	NA
Right	0.659 (0.154‐2.810)	.57
Pre‐TAE tumor volume (per 1 cm^3^)	1.002 (0.999‐1.005)	.10
Tumor volume/body weight (cm^3^/kg)	1.058 (1.014‐1.103)	.01^†^
Signs/history		
History of signs of gastrointestinal disease+ (vs −)	2.725 (0.556‐13.34)	.21
History of abdominal effusion+ (vs −)	3.432 (0.797‐14.79)	.10
History of weight loss+ (vs −)	1.702 (0.447‐6.476)	.44
History of hemoabdomen+ (vs −)	4.909 (1.135‐21.22)	.03^†^
History of jaundice+ (vs −)	3.609 (0.374‐34.86)	.27
Embolic agent		
Embosphere (vs Gelpart)	0.465 (0.124‐1.746)	.26

Abbreviation: TAE, transcatheter arterial embolization.

^†^ *P* < .05.

### 3.3 Secondary endpoint

**FIGURE 5:**
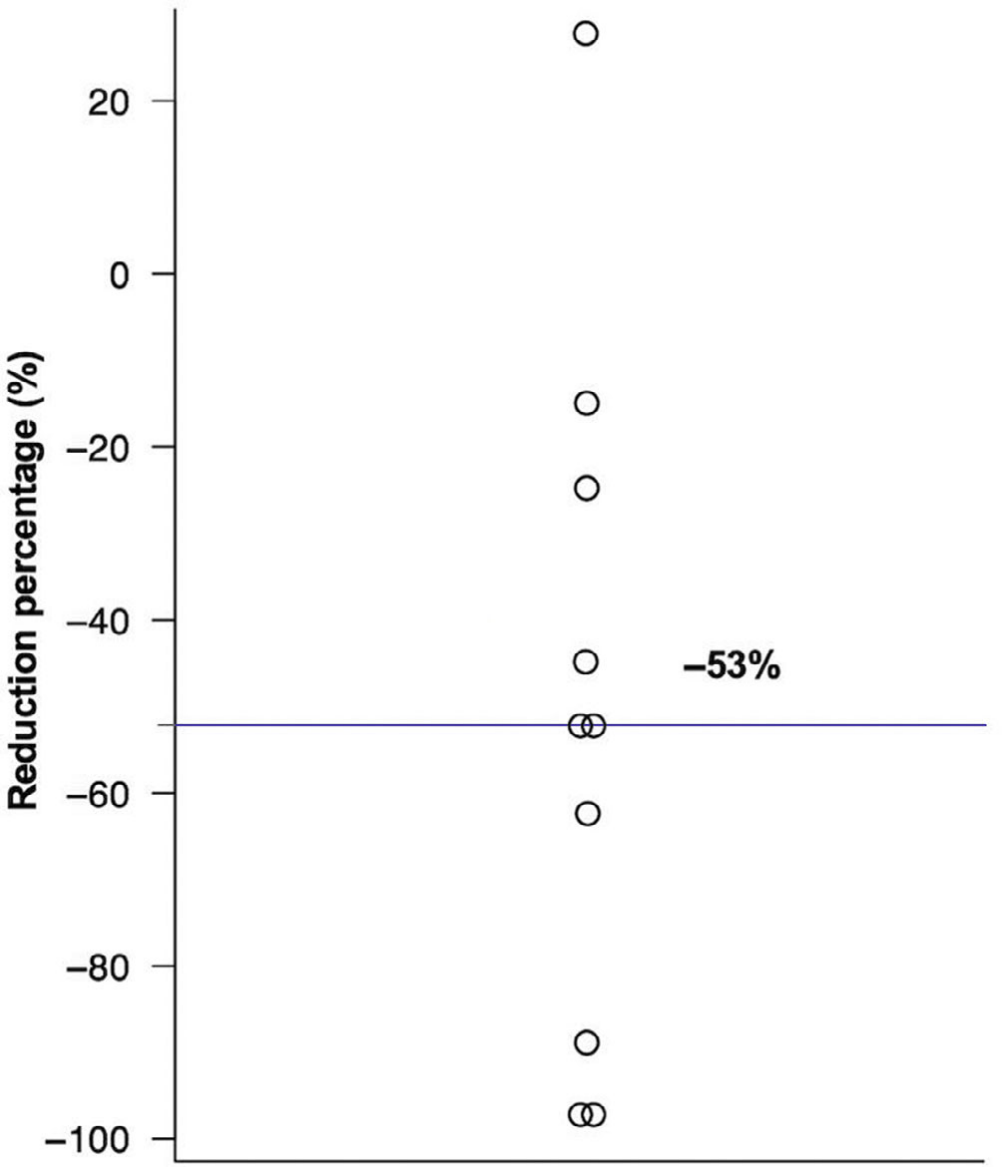
Dot plot of the tumor reduction percentage in 10 dogs undergoing computed tomography examination 1 to 2 months after transcatheter arterial embolization. The median reduction percentage is −53%.

**FIGURE 6:**
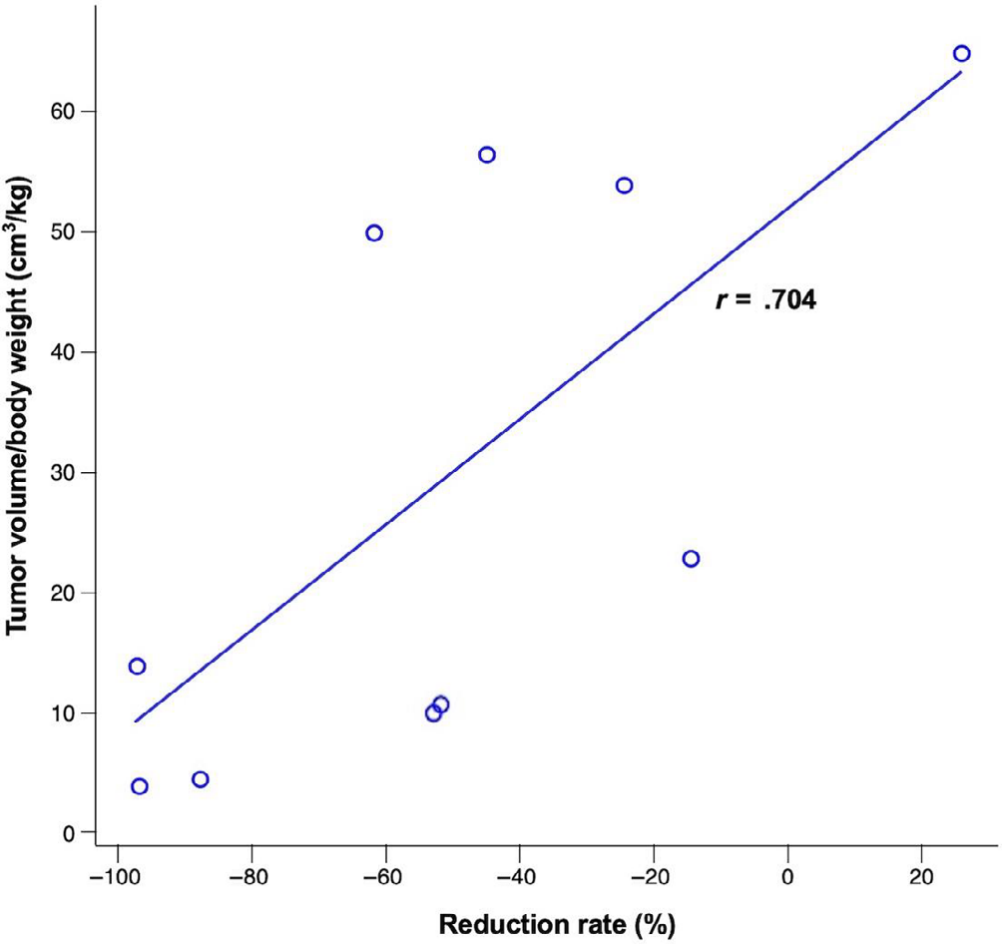
Scatter plot of the relationship between tumor volume/body weight ratio and tumor reduction percentage. The correlation coefficient of 0.704 indicates that there is a positive correlation between tumor volume/body weight ratio and reduction percentage.

In 1 case, the mass increased in size after TAE, but the others shrank. The average tumor reduction percentage of all 10 cases was −51% ± 40%, and the median reduction percentage was −53% (Figure 5). A univariate linear regression analysis of the predictive factors for shrinkage was performed using the independent variables stated in Section 2.7, and the results showed that the pre‐TAE tumor volume/body weight ratio was significantly correlated with shrinkage (regression coefficient: 1.133, 95% CI: 0.200‐2.066, *P* = .02; Table 4). Figure 6 shows a scatter plot of the relationship between tumor volume/body weight ratio and the reduction percentage which was significant in the linear regression analysis. The correlation coefficient of 0.704 indicates that there is a positive correlation between tumor volume/body weight ratio and shrinkage.

**TABLE 4:** Univariate linear regression model for predicting tumor reduction volume in 14 dogs undergoing TAE for hepatocellular masses.

Variable	Regression coefficient (95% confidence interval)	*p* value
Dog factors		
Age (per 1 year)	−0.840 (−2.208 to 0.528)	.12
Weight (per 1 kg)	−0.654 (−8.539 to 7.231)	.85
Tumor factors		
Hepatic segment (vs left)		
Center	−60.06 (−162.44 to 42.32)	.21
Right	−17.41 (−80.11 to 45.29)	.53
Pre‐TAE tumor volume (per 1 cm^3^)	0.066 (−0.041 to 0.174)	.19
Tumor volume/body weight (cm^3^/kg)	1.133 (0.200 to 2.066)	.02^†^
Signs/history		
History of signs of gastrointestinal disease+ (vs −)	−4.78 (−65.79 to 56.31)	.86
History of abdominal effusion+ (vs −)	31.06 (−30.69 to 92.81)	.28
History of weight loss+ (vs −)	−37.41 (−96.78 to 21.95)	.18
History of hemoabdomen+ (vs −)	47.83 (−17.95 to 113.6)	.13
History of jaundice+ (vs −)	NA	NA
Embolic agent		
Embosphere (vs Gelpart)	19.68 (−55.09 to 94.44)	.56

Abbreviation: TAE, transcatheter arterial embolization.

^†^ *p* < .05.

**FIGURE 7:**
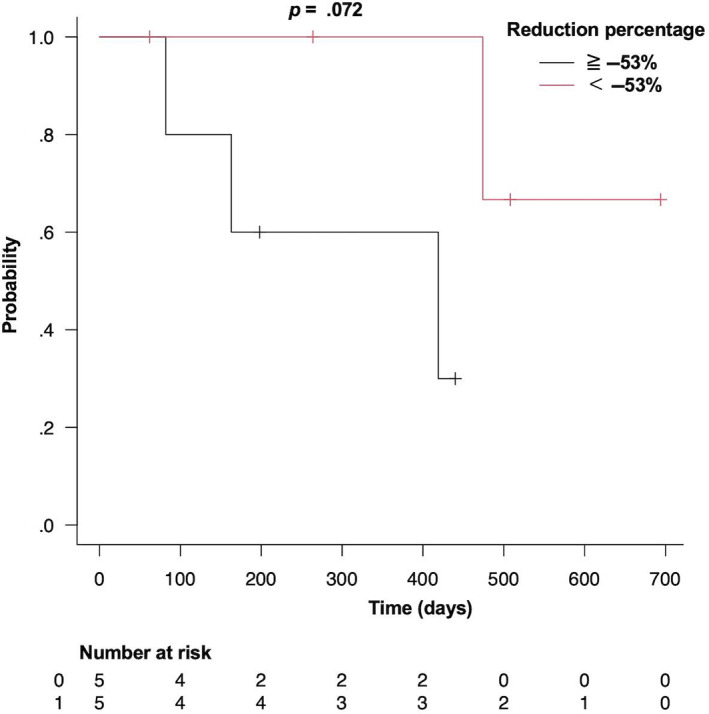
Kaplan‐Meier survival curves of 10 dogs with a tumor reduction percentage of ≥−53% (black) and <−53% (red).

A log‐rank test was performed to analyze the relationship between the tumor reduction percentage and survival time by dividing the dogs into two groups based on whether their reduction percentage was ≥−53% or <−53%. There was no significant difference between the groups (*P* = .07; Figure 7).

### 3.4 Complications

After TAE, 7 of the 14 dogs had minor complications thought to be postembolization syndrome. Signs of postembolization syndrome, as classified by the Veterinary Cooperative Oncology Group‐Common Terminology Criteria for Adverse Events version 2,^22^ included vomiting (Grade 1: 2/14, 14%; Grade 2: 1/14, 7%), diarrhea (Grade 1: 1/14, 7%; Grade 2: 1/14, 7%), and hyporexia (Grade 1: 7/14, 50%). All cases of postembolization syndrome improved within 3 days with intravenous fluid therapy and symptomatic treatment.

As a major complication, there were two cases of perioperative death that resulted from TAE. One dog died on the first postoperative day. In this case, the tumor involved the caudal vena cava, and surgical resection was associated with a high risk. Therefore, the dog was treated with supportive care including ascites aspiration and fluid therapy for ~1 month after the initial CT scan. However, ascites repeatedly accumulated after aspiration. TAE was performed, but the dog died the next day. Since significant azotemia (blood urea nitrogen = 138 mg/dL), hyperphosphatemia (8.2 mg/dL), and hyperkalemia (6.6 mmol/L) were observed after TAE, the cause of death was suspected to be tumor lysis syndrome and associated acute renal failure. The other dog died on the 15th postoperative day. This case showed postembolization syndrome after TAE with fever and inappetence, which improved after the intravenous administration of lactated Ringer's solution, and the dog was discharged on the third postoperative day. After discharge, the dog's condition remained stable, but most of the tumor was necrotized by TAE, resulting in a cyst. On the 15th day after TAE, the cyst ruptured suddenly, causing intra‐abdominal bleeding and collapse. The dog died of hypovolemic shock.

## 4 DISCUSSION

In this study, we analyzed the outcomes of dogs that underwent TAE for primary hepatocellular masses, and the tumor reduction percentage calculated using CT. The following findings were identified: First, in our cohort of dogs with hepatocellular masses that underwent TAE, a history of intra‐abdominal hemorrhage and the tumor volume/body weight ratio were potential prognostic factors of outcome. Second, we identified that tumor volume/body weight ratio was the most promising predictor of shrinkage and could be a predictor of treatment response.

In human medicine, TAE was first reported in 1983.^23^ Since then, it has become a common treatment option for hepatocellular carcinoma. Subsequently, intra‐arterial chemotherapy was added to TAE, introducing transcatheter arterial chemoembolization (TACE) as a treatment option. The survival time of  patients that have undergone TACE or TAE is significantly improved compared with that of those receiving symptomatic treatment.^24^, ^25^, ^26^ In human medicine, TACE and TAE tend to be indicated in cases with 4 or more nodules or three or fewer nodules resistant to resection or thermal ablation.^15^ However, TACE and TAE have had positive effects and achieved a certain level of effectiveness in cases where other treatments were indicated in the guidelines.^24^, ^25^, ^26^ Indications for TACE or TAE are determined by tumor vascularity, tumor location, the presence of portal vein tumor thrombus, and the degree of liver dysfunction.^24^, ^25^, ^26^ In human medicine, TAE tends to be performed for unresectable, large hepatocellular carcinomas, and it might have fewer adverse effects than TACE.^6^

In veterinary medicine, limited treatment options are available for liver tumors.^4^, ^5^ Surgery is the first‐line treatment, but there are cases in which surgery is inappropriate or the owner does not wish for their pet to undergo surgery. In such cases, TAE or TACE is an option. In 2002, arterial embolization procedures were performed in three dogs and one goat for the first time in veterinary medicine, two of which were TACE procedures for malignant liver tumors.^16^ In this study, PVA granules were used as the embolic agents. In 2003, TACE procedures using iodized oil combined with chemotherapy were performed in two dogs.^17^ In 2015, TAE procedure was performed in one cat with hepatocellular carcinoma,^18^ and TAE procedure was performed in a cat with cholangiocellular adenoma with intra‐abdominal hemorrhage in 2021.^19^ In 2020, a prognostic survey of TACE using drug‐eluting beads in dogs was conducted. The post‐treatment MST was 337 days (range, 22‐1061), with a significantly poorer prognosis in dogs with a history of weight loss than in those without.^27^

The embolic agents used in this study, Gelpart and Embosphere, are frequently used for TAE in human and veterinary medicine. There are several basic studies on these embolic agents in veterinary medicine using normal dogs. In one study, Gelpart was injected into the left hepatic artery of five normal beagle dogs, followed by observation for 12 weeks.^28^ Although transient increases in liver enzymes were observed, no major complications occurred, concluding that TAE using Gelpart could be safely indicated for hepatocellular carcinoma in dogs. In another study, Embosphere was injected into the left hepatic artery of 4 normal beagle dogs and CT examination, biochemical analysis, and histological examination were performed over a 12‐week observation time.^29^ Postoperative CT revealed consistent embolization of the hepatic artery during the experimental period in three dogs. Liver enzyme levels increased slightly after embolization but decreased within a normal range. Therefore, it is concluded that selective TAE using Embosphere is well tolerated in normal dogs and could be applicable to canine hepatocellular carcinoma.

In the present study, the MST of 14 dogs with hepatocellular masses that underwent TAE was 419 days, and the 1‐year survival rate was 55%. A previous report has suggested that the survival time of dogs with hepatocellular carcinoma that remains untreated is ~9 months, indicating that TAE could extend survival.^4^ This result could justify presenting TAE as a minimally invasive treatment option for dogs with hepatocellular masses.

A history of intra‐abdominal hemorrhage before TAE has a significant correlation with prognosis, indicating that it could be a predictive factor of outcome. This could be because in cases where the tumor has ruptured, there is a high possibility of rebleeding after TAE.^30^, ^31^, ^32^ In veterinary medicine, we often encounter large, supcapsular, and exophytic tumors. There are many cases in which hepatic masses with such characteristics achieve spontaneous hemostasis after rupturing and causing intra‐abdominal bleeding; however, such cases have a high risk of rebleeding, which might be further induced by necrosis of the tumor after TAE. In addition, poor general condition because of decreased liver function and abnormal blood coagulation because of large liver masses might also be related.^33^, ^34^ However, a previous study reported that intra‐abdominal hemorrhage before TAE was not a prognostic factor,^27^ which differs from the findings of this study. In that study, there were only three cases with a history of bleeding, which might be because of slightly different cohort, as well as protocol differences, such as the type of embolization agent used and whether anticancer drugs were used simultaneously. Whether intra‐abdominal hemorrhage is truly a prognostic factor of TAE or TACE requires further investigation in larger studies.

Pre‐TAE tumor volume/body weight ratio was also a significant prognostic factor of outcome, and the results showed that larger tumor volume/body weight ratios were associated with shorter survival time. This is the same result as previous studies.^35^ In human medicine, this is considered to be related to blood flow inside the tumor; that is, as the tumor grows, the area with poor internal blood flow increases, and the effect of TAE tends to be limited.^36^, ^37^, ^38^, ^39^ In addition, as the tumor grows, the number of feeding blood vessels increases. Therefore, because TAE cannot block all blood flow, some tumor tissue remains alive, and the tumor might begin to grow again in the early postoperative period.^38^, ^39^ This is considered to be one of the reasons why the tumor continued to grow after TAE in one dog. The increased risk of tumor lysis syndrome in cases with a large tumor volume/body weight ratio, such as that of the dog that died the day after TAE, might also contribute to poor outcomes. No correlation was found between embolic agent type and outcome in this study.

The average tumor reduction percentage calculated using CT scans 1 to 2 months after TAE was −51%, which had a significant positive correlation with pre‐TAE tumor volume/body weight ratio. This is likely because the proportion of necrotic tissue contained inside the tumor increases as the mass grows, and blood flow does not reach the inside, as is reported in human medicine.^36^, ^37^, ^39^ The reduction percentage also did not correlate with the type of embolization agent used in this study.

In human medicine, patients with a good response to initial TAE are considered to have a good prognosis because they are less susceptible to tumor side effects postoperatively.^36^, ^37^, ^38^, ^39^ In the present study, no significant difference was observed between the two groups divided based on the median tumor reduction percentage in the survival analysis (*P* = .07). This might be because of a type II statistical error caused by a small number of cases.

Major complications in this case group included those seen in the dog which died on the first day after TAE and the dog that died on the 15th post‐TAE day. The cause of death in the first dog was considered to be tumor lysis syndrome, and this dog had a tumor volume/body weight value of 107.9 cm^3^/kg, which was the largest among all 14 dogs. Tumor lysis syndrome is a condition that causes hyperkalemia, hyperphosphatemia, hyperuricemia, and renal dysfunction because of acute necrosis of a large amount of tumor tissue.^40^ Dogs with extremely large tumors are at a high risk of tumor lysis syndrome and might require careful consideration of TAE.^40^, ^41^ Moreover, as seen in the second dog, a large tumor might become necrotic and cystic after TAE, so the condition of the tumor should be monitored frequently by ultrasonography after TAE. If a large cyst develops inside, aspiration of the contents could be considered.^41^, ^42^

Limitations of the present study included the small number of cases and the use of univariate analysis.

This study provides promising data on the outcomes and tumor reduction of dogs that underwent TAE for primary hepatocellular masses. The MST of the 14 dogs with hepatocellular masses that underwent TAE was 419 days, and the 1‐year survival rate was 55%; therefore, TAE could prolong survival time compared with that of dogs left untreated although there was no group of untreated dogs in this study. In the prognostic factor analysis, a history of intra‐abdominal hemorrhage and tumor volume/body weight ratio were significantly correlated with outcome and could be important prognostic factors for outcome after TAE. The average tumor reduction percentage was −51% and the pre‐TAE tumor volume/body weight ratio was the most strongly correlated factor, indicating that it could be a predictor of therapeutic response.

## CONFLICT OF INTEREST DECLARATION

Authors declare no conflict of interest.

## OFF‐LABEL ANTIMICROBIAL DECLARATION

Authors declare no off‐label use of antimicrobials.

## INSTITUTIONAL ANIMAL CARE AND USE COMMITTEE (IACUC) OR OTHER APPROVAL DECLARATION

Authors declare no IACUC or other approval was needed.

## HUMAN ETHICS APPROVAL DECLARATION

Authors declare human ethics approval was not needed for this study.
